# In-Hospital Cardiac Arrest (IHCA) and Outcomes in Patients Admitted With COVID-19 Infection

**DOI:** 10.7759/cureus.15365

**Published:** 2021-06-01

**Authors:** Rahul Khosla, Joseph Delio, Lisa N Glass, Shikha G Khosla, Omar Awan, Amandeep Bawa, Kavita Vyas

**Affiliations:** 1 Pulmonary and Critical Care Medicine, Veterans Affairs Medical Center, Washington, USA; 2 Pulmonary and Critical Care Medicine, The George Washington University School of Medicine and Health Sciences, Washington, USA; 3 Endocrinology, Diabetes and Metabolism, Veterans Affairs Medical Center, Washington, USA

**Keywords:** covid 19, in hospital cardiac arrest, critical care and hospital medicine, cardiac arrest outcome, veterans affairs

## Abstract

During the COVID-19 pandemic, many patients are hospitalized, and those suffering from in-hospital cardiac arrest (IHCA) have been previously reported to have poor outcomes. This is a single-center, retrospective, observational study conducted at the Veterans Affairs Medical Center, Washington, DC, USA. The inclusion criteria were: patients admitted to the hospital with a diagnosis of COVID-19 who underwent cardiopulmonary resuscitation (CPR) for IHCA. Patients were labeled as COVID-19 positive based on a laboratory-confirmed positive polymerase chain reaction test. Patients with do-not-resuscitate (DNR) orders, those who were made comfort care, or enrolled in hospice were excluded. The study was approved by the hospital’s institutional review board. A total of 155 patients with COVID-19 infection were admitted; 145/155 (93.5%) admitted to the medical floor and 10/155 (6.5%) to the medical intensive care unit (MICU). 36/145 (24.8%) floor patients were upgraded to MICU. Of the 46 patients treated in MICU, 17/46 (36.9%) were excluded for DNR status. From the remaining 29/46 (63.1%) patients, 19/29 (65.5%) patients survived, and 10/29 (34.5%) patients had IHCA. All 10/10 (100%) died after CPR without return of spontaneous circulation (ROSC). The initial rhythm was non-shockable in all patients, with pulseless electrical activity (PEA) in 7/10 (70%) and asystole in 3/10 (30%) patients. Patients with COVID-19 infection who had an IHCA and underwent CPR had a 0% survival at our hospital. Discussions on advanced care options, especially CPR, with COVID-19 patients and their families, are important as the overall prognosis after CPR for IHCA is poor.

## Introduction

The year 2020 witnessed a global pandemic with an outbreak of a novel coronavirus (COVID-19) that spread widely across the world as different epicenters emerged during the year. As we write the paper, the World Health Organization (WHO) is reporting more than 83 million confirmed cases worldwide with 1.8 million deaths [1]. COVID-19 has varied in its clinical presentation from asymptomatic carriers and mild infection to severe multi-system organ failure and death [2]. Hospitalized patients tend to be older with a high mortality rate [3]. Prior to the pandemic, in-hospital cardiac arrest (IHCA) has been reported in 1.2% of hospitalized patients numbering close to 290,000 adults each year in the United States [4]. With a mean age of 66 years, 58% being men, and the most common presenting with rhythm (81%) that is non-shockable (i.e., asystole or pulseless electrical activity [PEA]) [4]. The cause of the cardiac arrest is most often cardiac (50%-60%), followed by respiratory insufficiency (15%-40%) [4]. Survival after IHCA has improved over the last decade, probably related to a concerted effort of improved education, quality care measures, and medical care [5]. COVID-19 brought many challenges when caring for hospitalized patients, as a major concern has been the protection of healthcare workers from exposure and transmission of infection. Despite following American Heart Association (AHA) advanced cardiac life support (ACLS) and cardiopulmonary resuscitation (CPR) guidelines, there have been studies showing high mortality from COVID-19 [3,6,7]. In this study, we aimed to investigate the outcome after IHCA in COVID-19 patients at our hospital.

## Materials and methods

Study design and setting

This is a single-center, retrospective, observational study conducted at the Veterans Affairs Medical Center, Washington, DC, United States, between March 1, 2020 and August 15, 2020. Patients admitted to the hospital with a diagnosis of COVID-19 who underwent CPR for IHCA were included in the study. Patients were defined as COVID-19 positive if they had a laboratory-confirmed positive polymerase chain reaction (PCR) test. Patients with do-not-resuscitate (DNR) orders, those who were given comfort care, or enrolled in hospice were excluded from the study. The study was approved by the institutional review board of the hospital, and the requirement of informed consent was waived. Veterans Affairs Medical Center, Washington, DC, is a 150-bed teaching hospital with a 12-bed medical intensive care unit (MICU), 12-bed surgical intensive care unit (SICU), and a 12-bed step-down unit. The intensive care units (ICUs) are closed units under the care of intensivists, and all floor-level patients are under the care of dedicated hospitalists. The operations of the hospital have been modified during the COVID-19 pandemic to create a 30-bed isolation medical ward with negative pressure rooms for patients with COVID-19 infection requiring up to 6 liters/minute of oxygen by nasal cannula. Higher acuity with COVID-19 infection, including those requiring oxygen via high flow nasal cannula (HFNC), non-invasive or invasive mechanical ventilation, or renal replacement therapy, were cared for in the 12-bed step-down unit and the 12-bed MICU. A code blue team is available 24/7 and directs ACLS across the hospital. The team is alerted by the pager system. The process has been amended during the COVID-19 pandemic in the following manner. First, tracheal intubations are done only by anesthesiologists for patients diagnosed or suspected of COVID-19 infection. Next, responders to code blue are to ensure that proper personal protective equipment (PPE) is worn by every member of the team despite a potential delay in response. PPE includes a gown, gloves, an N-95 mask, and a face shield. Finally, the number of personnel in the room is restricted and includes the following: anesthesiologist for the airway, two persons for chest compressions who alternate every two minutes, a nurse for delivering medications, a nurse as a recorder, and a communicator with a two-way radio to communicate needs with the main nursing station. Besides these modifications, resuscitation followed CPR guidelines from AHA.

Data collection

Demographic, clinical, and CPR data of patients with COVID-19 pneumonia were collected from the electronic medical record using the Utstein style guidelines. Information regarding patients' age, sex, race, location at the time of cardiac arrest, comorbid illnesses, an initial rhythm at the time of cardiac arrest, and time to return of spontaneous circulation (ROSC) were collected and documented into an electronic database. The primary outcome was an immediate survival with ROSC, and the secondary outcome was 30-day survival.

Statistical methods

Categorical variables are shown as numbers and percentages in each category. Continuous variables are reported as means with standard deviation (SD) or medians and interquartile ranges (IQRs). A Chi-square test was used to examine the baseline differences in demographics and clinical characteristics. A P-value < 0.05 was considered statistically significant. Statistical analysis was performed using MedCalc statistical software (2020 MedCalc Software Ltd., Ostend, Belgium).

## Results

**Figure 1:**
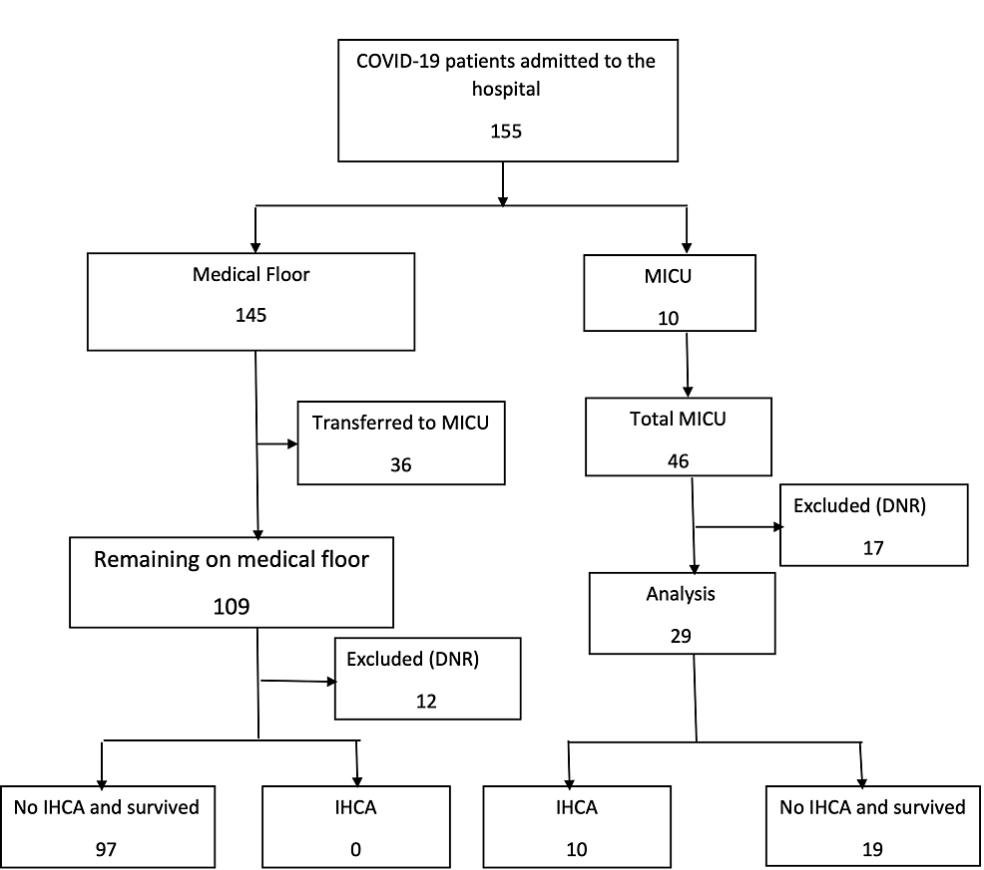
Flow diagram showing the number of COVID-19 patients admitted to the hospital with their location and outcome Abbreviations: DNR, do-not-resuscitate; IHCA, in-hospital cardiac arrest; MICU, medical intensive care unit.

From March 1, 2020 to August 15, 2020, a total of 155 patients with COVID-19 infection were admitted to the Veterans Affairs Medical Center, Washington, DC. A total of 145/155 (93.5%) patients were initially admitted to the medical floor under the care of hospitalists, and 10/155 (6.5%) patients to the MICU under the care of intensivists. Of those, 36/145 (24.8%) patients admitted to the medical floor were transferred to MICU for worsening clinical status during their hospital course. Of the 46 patients treated in MICU, 17/46 (36.9%) were either DNR prior to admission or were made so during their hospital course and were excluded from the analysis. Of the remaining 29/46 (63.1%) patients, 19/29 (65.5%) patients survived and 10/29 (34.5%) patients had IHCA. All 10/10 (100%) died after CPR without ROSC. This is detailed in Figure 1. The initial rhythm was non-shockable in all 10/10 (100%) patients, with PEA in 7/10 (70%) patients and asystole in 3/10 (30%) patients. The median duration of CPR was 18 minutes (IQR, 11-26 minutes). The survival to discharge was 0/10 (0%) patients. The median number of days in MICU was eight (IQR, 3.35-10 days), and the median number of days from admission to IHCA was eight (IQR, 4.25-10.75 days). A total of 109/145 (75.2%) patients were treated by the hospitalists on the medical floor, of which 12/109 (11%) were either DNR before admission or made DNR during the hospital course. Of those treated, 106/109 (97.2%) patients survived, and 3/109 (2.8%) died. The three patients were DNR, and none of the patients on the medical floor had IHCA. The median age of the patients with IHCA was 69.3 years (IQR, 65.75-72.75), nine patients (90%) were African American, and one patient (10%) was Caucasian. Patients' demographic characteristics, comorbidities, and CPR characteristics are summarized in Table 1. Diabetes mellitus was present in eight patients (80%), hypertension in six patients (60%), obesity in six patients (60%), obstructive sleep apnea in three patients (30%), coronary artery disease in three patients (30%), and peripheral vascular disease in three patients (30%). At the time of cardiac arrest, 7/10 (70%) patients were receiving mechanical ventilation, 7/ 10 (70%) patients vasopressor support, and three patients (30%) renal replacement therapy. Anticoagulation for deep vein thrombosis prevention was given to nine patients (90%). One patient received azithromycin, hydroxychloroquine, and steroids; one patient received azithromycin, hydroxychloroquine, and tocilizumab; two patients received hydroxychloroquine and azithromycin; two patients received tocilizumab and azithromycin; two patients received steroids alone. Table 2 summarizes the list of medications the patients received.

**Table 1:** Patient demographics characteristics, comorbidities, and CPR characteristics Abbreviations: CAD, coronary artery disease; CHF, congestive heart failure; CKD, chronic kidney disease; CPR, cardiopulmonary resuscitation; CVA, cerebrovascular accident; ICU, intensive care unit; IQR, interquartile range; PVD, peripheral vascular disease; ROSC, return of spontaneous circulation; SOFA, sequential organ failure assessment.

Characteristic	No. (%)
Sex	
Male	10/10 (100)
Female	0/10 (0)
Ethnicity	
African American	9/10 (90)
White	1/10 (10)
Age, median (IQR), y	71 (65.75-72.75)
Comorbidities	
Hypertension	8/10 (80)
Diabetes Mellitus	6/10 (60)
Obesity	6/10 (60)
CAD	3/10 (30)
CHF	3/10 (30
PVD	3/10 (30)
CKD	2/10 (20)
CVA	1/10 (10)
Cirrhosis	1/10 (10)
CPR initial rhythm	
Pulseless electrical activity	7/10 (70)
Asystole	3/10 (30)
Achieved ROSC	0/10 (0)
Duration of CPR, median (IQR), min	18 (11-26)
Survival to discharge	0/10 (0)
ICU length of stay, median (IQR), days	8 (3.25-10)
SOFA, median (IQR)	5 (4-5)

 

**Table 2:** List of medications received by the patients Steroids: one patient received methylprednisolone 40 mg twice daily with taper, and another patient received methylprednisolone 125 mg three times daily for three doses.

Medications	No.
Steroids	2
Tocilizumab and Azithromycin	2
Hydroxychloroquine and Azithromycin	2
Azithromycin, Hydroxychloroquine, and Tocilizumab	1
Azithromycin, Hydroxychloroquine, and Steroids	1
Deep vein thrombosis prophylaxis	9

## Discussion

Prior to the COVID-19 pandemic, large variations in hospital-wide incidence rates of adult IHCA have been reported, ranging from 3.8 to 13.1 per 1,000 admissions, along with a variation in survival to discharge rate, ranging from 10% to 20% [8,9]. Recent studies have reported poor outcomes after IHCA in patients with COVID-19 infection [3,6,7]. In our study, 10 of the 46 patients admitted to the MICU suffered IHCA with a 100% mortality and 0% ROSC following CPR. The initial rhythm was non-shockable for all 10 (100%) patients, with PEA (7/10, 70%) being the most common. Similar to our findings, Thapa et al. from Michigan reported a 100% mortality rate following CPR for IHCA in 54 patients with COVID-19, with PEA being the most common initial rhythm in 44/54 (81.5%) patients [6]. Miles et al. compared the outcomes of IHCA at their hospital in New York during the COVID-19 pandemic with a pre-COVID-19 period. They reported a survival rate of 3% during the COVID-19 pandemic compared to a 13% survival rate (P = 0.007) in the pre-COVID-19 period, where PEA was the most common initial rhythm (67/125, 54%) [7]. Among 136 patients with IHCA in a study from Wuhan, China, 18 patients (13.2%) achieved ROSC, four (2.9%) patients survived at least 30 days, and one patient had a favorable neurological outcome at 30 days; the most common initial rhythm was asystole (122/136, 89.1%) [3,9]. Mortality in patients with COVID-19 infection has varied across the world and is higher in men, older patients, and those with comorbid illnesses [2,10,11]. Additional studies have shown improvement in achieving ROSC after IHCA in COVID-19 [12-14]. Mitchell et al. reported a 22% rate of ROSC after CPR in COVID-19 patients in a study of 260 IHCA including 11 medical centers [12]. They noted large variability of ROSC rate between medical centers, suggesting that single-center studies may not be representative. Yuriditsky et al. compared IHCA in 55 COVID-19 patients to 55 patients in 2019 and found the rate of ROSC after IHCA was not significantly different; 38.2% in COVID patients compared to 49.1% (P=0.336) [13]. Sultanian et al. reported 39.5% 24-hour survival of COVID-19 patients after IHCA in the Swedish Registry for Cardiopulmonary Resuscitation [14]. They compared the adjusted probability of 30-day survival after IHCA in COVID-19 patients (23.1%), patients without COVID-19 during the pandemic (39.5%) and pre-pandemic (36.5%).

On reviewing the data from three studies on outcomes in COVID-19 patients with IHCA, the incidence of IHCA was higher in men, patients above 60 years of age, those with diabetes mellitus and hypertension [3,6,7]. In our study, all COVID-19 patients with IHCA were men, the mean age was 70 years; diabetes mellitus, hypertension, and obesity were the most common comorbid illnesses. During the study period, 46 patients with COVID-19 infection were admitted to the MICU, of which 27 died (58.9%) died, 17 patients had prior DNR orders or were made DNR during their hospital course, and all of them expired. The cause of high mortality in COVID-19 patients post-IHCA has been speculated in other studies. These causes include lack of medical resources, uncertain quality of CPR, severe systemic illness with multi-organ dysfunction in COVID-19 patients, lack of continuous oximetry monitoring on general wards resulting in delayed detection of rapid respiratory failure, and a delay in response secondary to PPE requirements have been implicated as a reason for high mortality [3,6,7]. All patients in our study who had IHCA had continuous monitoring of their vitals in the MICU at the time of the cardiac arrest. They were all severely ill with an average sequential organ failure assessment (SOFA) score of 4.9 on admission. Among these patients, 7/10 (70%) were already intubated and on mechanical ventilation, 7/10 (70%) were on vasopressors and 3/10 (30%) on renal replacement therapy. The requirement of PPE during intervention for cardiac arrest was stringent at our hospital to protect the staff, which may have led to a delay in response. Studies have shown that mortality increases by 10%-15% for every minute delay in response to cardiac arrest [15]. Our study period includes the early phase of the COVID-19 pandemic when information on anti-inflammatory, anticoagulation, anti-viral, and other therapies were not yet published. We now know that the use of steroids resulted in lower mortality in COVID-19 patients and the use of remdesivir shortens the time to recovery in adults who were hospitalized with COVID-19 infection [16,17]. Concern has been raised regarding CPR for COVID-19 patients, as studies show poor outcomes, and it increases exposure risk to healthcare personnel despite protective measures and protocols [3,6,7]. This is an ethical debate that needs to be conducted in the correct forum, and our study is not designed to address this. At no time at our institution were patients with COVID-19 infection made DNR without a discussion with the patient or a surrogate.

There are several limitations to our study. First, it is a retrospective analysis, which allows for confounding and selection bias. Second, it is a single-center study with a small number of patients; hence, the results cannot be generalized to other healthcare settings. Third, data on the timeliness, quality, and other specifics regarding CPR is lacking. The study period was in the earliest portion of the pandemic, and since that time, treatments have improved mortality.

## Conclusions

Patients with COVID-19 infection who had an IHCA and underwent CPR had a 0% survival at our hospital during the study period March 2020 to August 2020. During resuscitation, there is a chaotic environment that and healthcare personnel are placed at risk of contracting the virus despite wearing adequate PPE. Discussions on advanced care options, especially CPR, with COVID-19 patients and their families are important as the overall prognosis after CPR for IHCA is poor.
